# The diagnostic role of microRNA-34a in breast cancer: a systematic review and meta-analysis

**DOI:** 10.18632/oncotarget.15520

**Published:** 2017-02-20

**Authors:** Saber Imani, Xianqin Zhang, Hossein Hosseinifard, Shangyi Fu, Junjiang Fu

**Affiliations:** ^1^ Key Laboratory of Epigenetics and Oncology, The Research Center for Precision Medicine, Southwest Medical University, Luzhou, Sichuan, PR China; ^2^ Chemical Injuries Research Center, Baqiyatallah Medical Sciences University (BMSU), Tehran, Iran; ^3^ Research Center for Evidence Based Medicine (RCEBM), Tabriz University of Medical Sciences, Tabriz, Iran; ^4^ Honors College, University of Houston, Houston, TX, USA

**Keywords:** breast cancer, microRNA-34a, biomarker, diagnostics, meta-analysis

## Abstract

**Background:**

MicroRNA-34a (miR-34a) is a master regulator of tumor suppression in breast cancer (BC). This systematic review aims to analyze the diagnostic accuracy of miR-34a in the detection of BC as a biomarker.

**Results:**

A total of 1858 BC cases and 494 controls from thirteen eligible studies reported in 9 publications were included. The overall pooled sensitivity, specificity, negative likelihood ratio (NLR), positive likelihood ratio (PLR), and diagnostic odds ratio (DOR) were 85.50% (95% CI: 83.80-87.00%), 70.00% (95% CI: 65.80–74.10%), 0.29 (95% CI: 0.19–0.43), 2.58 (95% CI: 1.91–3.43), and 9.39 (95% CI: 5.47–16.12), respectively. Similarly, the overall area under the curve (AUC) of the summary receiver operating characteristic (SROC) was 0.80, indicating the high conservation of miR-34a as a biomarker. Furthermore, subgroup analysis suggested that the use of miR-34a as a biomarker is more accurate in tissue-based sample of invasive BC. We also indicated that miR-34a is a capable biomarker in diagnosing BC in people of Caucasian descent.

**Materials and Methods:**

A systematic search was conducted for eligible publications that address miR-34a expression level in BC cases and noncancerous controls. Diagnostic capacity of miR-34a for BC was assessed using pooled sensitivity and specificity, DOR, and AUC of SROC. PLR and NLR were verified to estimate the miR-34a diagnostic accuracy in clinical level. The quality of the included studies was assessed by QUADAS-2.

**Conclusions:**

These findings suggest miR-34a is a promising non-invasive biomarker in diagnosing BC. Well-designed cohort studies should be implemented to warrant the diagnostic value of miR-34a in clinical purposes.

## INTRODUCTION

Breast cancer (BC) is the second leading cause of mortality in females worldwide and the most frequently diagnosed cancer in the USA, estimated 14.6% (1.68 million) of all new cancer cases and 40,290 of all cancer-related deaths in 2015 [1, 2]. Since the BC is a genetically heterogeneous disease, clinical and diagnostic outcomes are widely disparate and routine clinic-pathological factors for diagnosis and/or prognosis of BC are potentially limited [3].

Certainly, early-stage detection and diagnosis of BC can reduce the mortality ratio, especially in some Asian-Pacific countries. Currently, mammogram screening for tissue- and serum-based tumor is the most effective screening tool for detecting the appearance or the recurrence of BC advancement [4, 5]. However, unavailability of hospital insurance, low sensitivity and specificity, high false positives, complexity, and high costs are main limitations of these diagnostic biomarkers to monitor disease progression or recurrence. For example, protein-based circulating tumor biomarkers, such as carbohydrate antigen 15–3 and tissue polypeptide specific antigen, are already applied in clinical diagnoses, but have low diagnostic sensitivity and specificity [6, 7]. Therefore, novel noninvasive diagnostic biomarkers with high sensitivity and specificity for early-stage BC detection are in great need [8].

MicroRNA-34a (miR-34a) represents a novel class of tumor suppressor miRNA, which negatively represses the oncogene expression by binding to the 3′-UTR of target mRNAs [9]. MiR-34a can antagonize many different oncogenic processes; inhibit tumor cell differentiation, proliferation, migration and invasion; and increase apoptosis and cell arrest. As evidenced by current literatures, miR-34a is found to be the mediator of tumor suppression by transcriptional regulating p53, NOTCH, epithelial-mesenchymal transition (EMT), and TGF-β signaling pathways [10–15]. Recent studies introduced miR-34a as a non-invasive urine-based biomarker for BC detection, with 61.0% sensitivity and 79.7% specificity [16–18]. Another study investigated the diagnostic accuracy of miR-34a by using fractionated radiation to create radiation-induced molecular targets [19]. A recently published study suggests that serum and plasma miR-34a levels were associated with the histologic grade of BC. However, there was no significant association between serum miR-34a expression and clinicopathologic features, such as hormone receptors and lymph node metastasis [16, 18]. Therefore, we conducted a comprehensive, systematic review and meta-analysis based on eligible studies to solve inconsistent and ambiguous findings and confirm the diagnostic value of miR-34a in BC. Furthermore, we planned to document the evidence for the use of miR-34a as a diagnostic marker to predict other clinical pathological features and outcomes of BC.

## RESULTS

### Literature search

**Figure 1:**
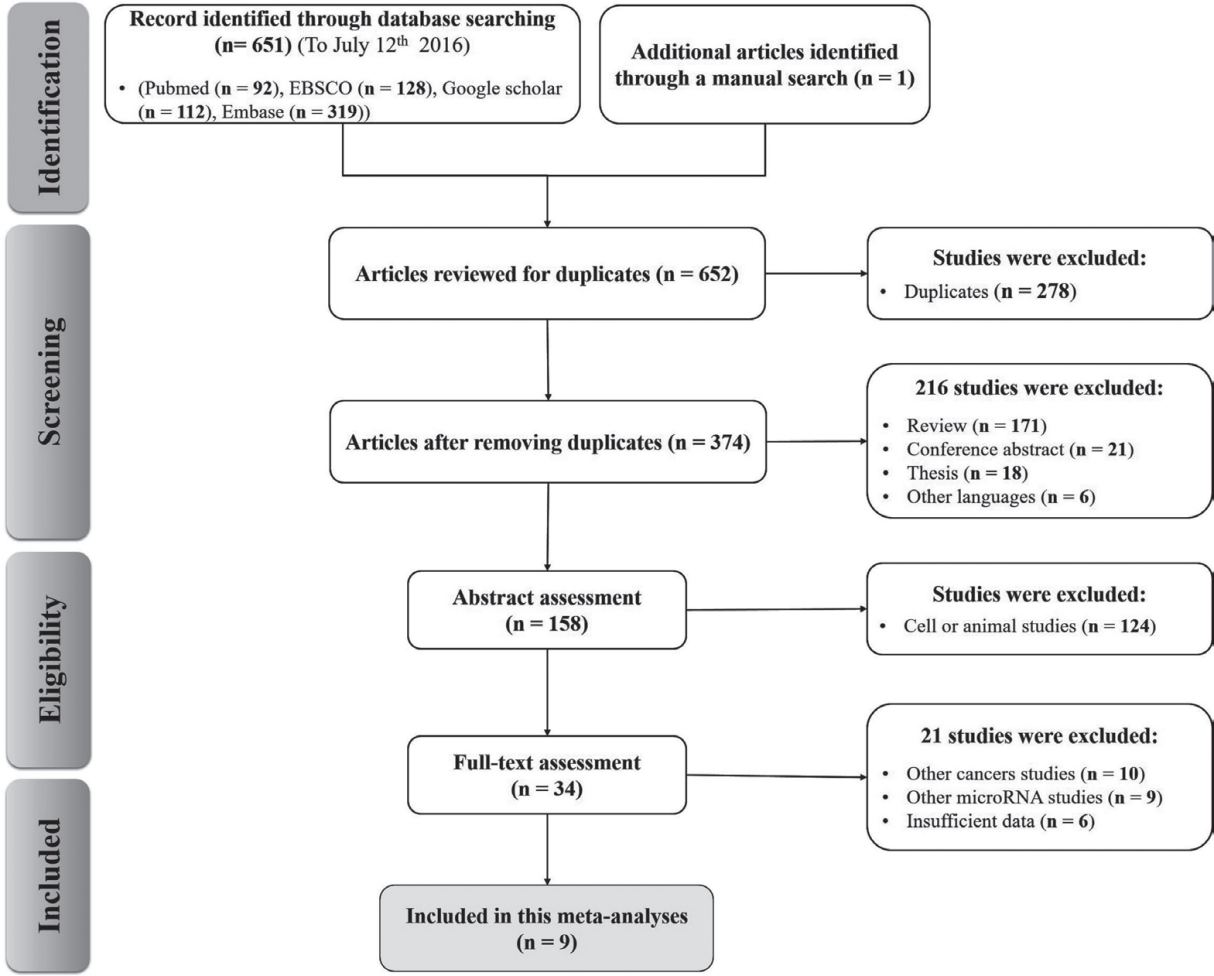
Flow chart of the study selection in the meta-analysis

A detailed flowchart of the screening and selection process in systematic reviews and meta-analyses (PRISMA) is shown in Figure 1. In total, 651 potentially eligible studies were obtained according to the inclusion and exclusion criteria from database searching and 1 record by manual search. Afterwards, 278 papers potentially eligible for exclusions were confirmed with the initial search strategy mentioned. Of the 374 candidate studies, 216 studies were excluded due to unrelated titles or abstracts while 158 articles were left for abstract assessment. After carefully reviewing titles and abstracts, 124 studies were precluded for obvious irrelevance because of cell or animal studies data. Of the remaining 34 full-text candidate articles, 21 potential studies were excluded, due to insufficient data or data concerning either other cancers or other microRNAs studies. Finally, 9 articles were considered in this meta-analysis [16–18, 20–25].

### Main results and quality assessment

**Figure 2:**
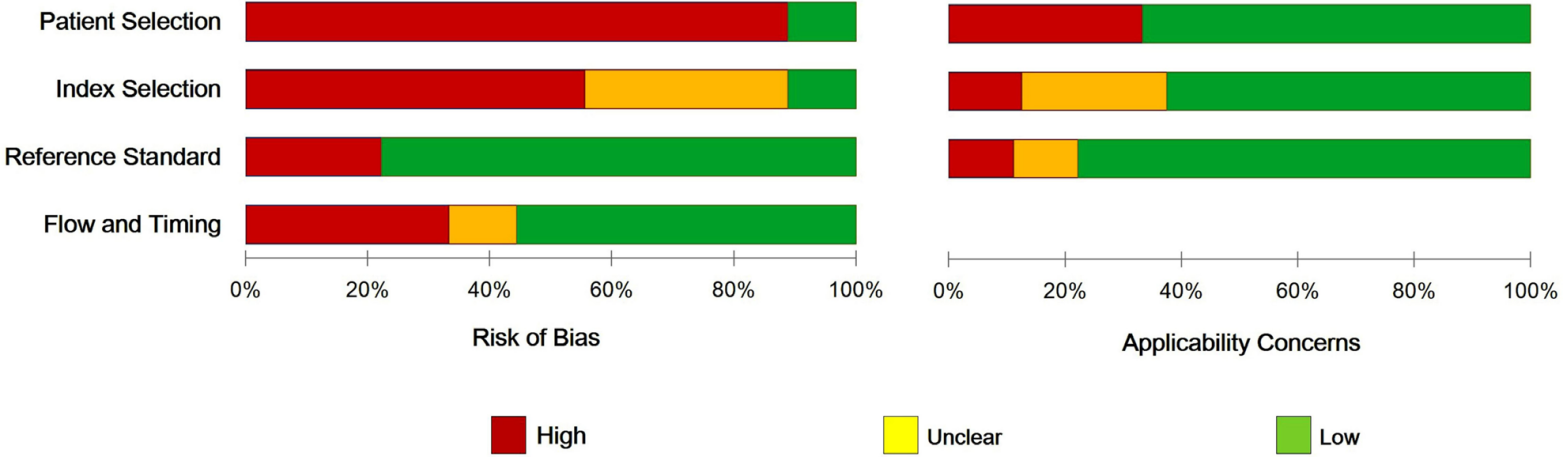
The QUADAS score, risk of bias and applicability concerns graph for quality assessment

The main clinical characteristics of the included studies were listed in Table 1 by order of quality assessment of diagnostic accuracy studies 2 (QUADAS-2) scores. Concerning the nine articles of interest, the first author, patient number, ethnicity, BC and sample type, characteristics of cases for each study as well as other necessary information were summarized. In total, 2352 subjects (1858 BC patients and 494 healthy controls) between 2010 and 2016 were included in our meta-analysis and histological classified into two types of BC (*n* = 13): invasive breast cancer (IBC, *n* = 7) and non-IBC (*in situ*) (*n* = 6). Of all the studies, 7 were focused on grade II/III BC (301 case) and 6 on grade I/II BC (1667 cases). MiR-34a expression levels were measured in formalin-fixed, paraffin-embedded tumor tissue (*n* = 5), serum (*n* = 3) and plasma (*n* = 1). While three studies used the *in situ* hybridization method, the quantitative real-time reverse transcription PCR (qRT-PCR) method was often used in the other studies to measure the expression of miR-34a by 2^−ΔΔCt^ method with different reference controls [17, 24]. Individually, the cut-off level of miR-34a appeared to be different (0.12–4.5) in different sample types. Notably, only two papers reported the sensitivity and specificity was directly extracted [18, 20]. QUADAS-2 results showed that no significant bias was presented in current meta-analyses (Figure 2). Detailed information of QUADAS-2 assessment is represented in Supplementary Table 1.

**Table 1:** Main characteristic of the included studies in this meta-analysis

Author (Ref.)	Year	Country	Ethnicity	BC type	Sample type	Sample size		Age		Diagnostic power				Cancer grade	Cut-off value	Genotyping method	Meas. type	Ref. control
						Case	Cont.	< 50	> 50	TP	FP	FN	TN					
Sanjay Mishra [18]	2015	India	Caucasian	Non-IBC	Plasma	45	45	NR	NR	NR	NR	NR	NR	ІІ/ІІІ	0.12	qRT-PCR	TaqMan	U6
Corinna Eichelser [20]	2013	Germany	Caucasian	Non-IBC	Serum	120	40	0	120	NR	NR	NR	NR	І/ІІ	1.02	qRT-PCR	TaqMan	miR-16
				IBC		32	40	0	32	NR	NR	NR	NR	ІІ/ІІІ	4.5			
Seema Agarwal [17]	2015	USA	Caucasian	IBC	Tissue	407	54	127	334	354	15	53	39	І/ІІ	NR	In situ hyb.	NR	NR
		USA				242	37	94	185	201	9	41	28					
		Poland				705	90	270	525	672	26	33	64					
Thalia Erbes [16]	2015	Germany	Caucasian	Non-IBC	Serum	24	24	24	24	19	6	5	18	І/ІІ	0.63	qRT-PCR	TaqMan	miR-16
Khan M.A. [21]	2016	China	Asian	IBC	Tissue	33	15	17	27	27	5	6	10	ІІ/ІІІ	0.22	qRT-PCR	TaqMan	U6
Imen Medimegh [22]	2014	Tunis	Caucasian	Non-IBC	Tissue	60	60	32	38	51	12	9	48	ІІ/ІІІ	1.45	qRT-PCR	SYBR	U6
Mei YiWu [23]	2014	China	Asian	Non-IBC	Tissue	42	18	NR	NR	33	7	9	11	ІІ/ІІІ	0.18	qRT-PCR	TaqMan	U6
Hanna Peurala [24]	2011	Finland	Caucasian	IBC	Tissue	59	13	406	766	46	4	13	9	ІІ/ІІІ	0.63	In situ hyb.	NR	NR
Carina Roth [25]	2010	Germany	Caucasian	Non-IBC	Serum	59	29	NR	NR	41	8	18	21	І/ІІ	1.02	qRT-PCR	TaqMan	NR
				IBC		30	29	NR	NR	19	8	11	21	ІІ/ІІІ	4.5			

Abbreviations: BC, breast cancer; IBC, invasive breast cancer; Non-IBC, non- invasive breast cancer; TP, true positive; FP, false positive; FN, false negative; TN, true negative; In situ hyb., in situ hybridization; qRT-PCR, quantitative real-time reverse transcription PCR; Meas., measurement; Ref., reference; U6, human U6 snRNA housekeeping small RNA controls; NR, not reported. All tissue samples are formalin-fixed, paraffin-embedded. “NR” parameters for the measurements type and reference control were considered as other categories. The fold changes in miR-34a expression were calculated using the 22^−ΔΔCt^ method. UD, unpublished data. Malignant tumors classified according the tumor-node-metastasis (TNM) stage.

### Quantitative synthesis

The primary results of meta-analysis on the expression of miR-34a and BC risk are shown in Table 2. There were no significant associations between miR-34a levels and BC susceptibility for all genetic models. An overall analysis between miR-34a and odds ratios (ORs) was performed and results showed that all studies exhibit moderate heterogeneity (I^2^ = 54.0%, *P* = 0.048). Then, a random effects model was applied to calculate a pooled OR and 95% confidence intervals (CIs), which were statistically significant in these cases (Table 2). Our results clearly showed heterogeneity of studies and analyses, so we then attempted to explain its sources from a randomized source of samples to calculate the accuracy of miR-34a. The threshold effect of spearman correlation coefficient is the main reason of heterogeneity in the test accuracy studies [26]. In this study, there was no heterogeneity from the threshold effect with the spearman correlation coefficient of sensitivity and 1-specificity of −0.415 (*P* = 0.158).

**Table 2:** Meta-analysis results for the expression of miR-34a and breast cancer risk

	No. of studies	Sample size (cases/controls)	x2	I2 (%)	Pooling Model	Pooled	OR (95%CI)	*P*-value
**Sensitivity**	13	1858/495	166.79	92.80	R	85.5	83.80–87.00%	0.001
**Specificity**	13	1858/495	34.03	64.70	R	70.00	65.80–74.10%	0.007
**PLR**	13	1858/495	48.49	75.30	R	2.58	1.94–3.44	0.001
**NLR**	13	1858/495	118.81	89.91	R	0.29	0.19–0.43	0.001
**DOR**	13	1858/495	49.90	76.00	R	9.39	5.47–16.11	0.002

Abbreviations: OR, odds ratio; CI, confidence interval; PLR, positive likelihood ratio; NLR, negative likelihood ratio; DOR, diagnostic odds ratio; x2, chi-squared; R, randomize model.

### Meta-analysis results

### Diagnostic accuracy

**Figure 3:**
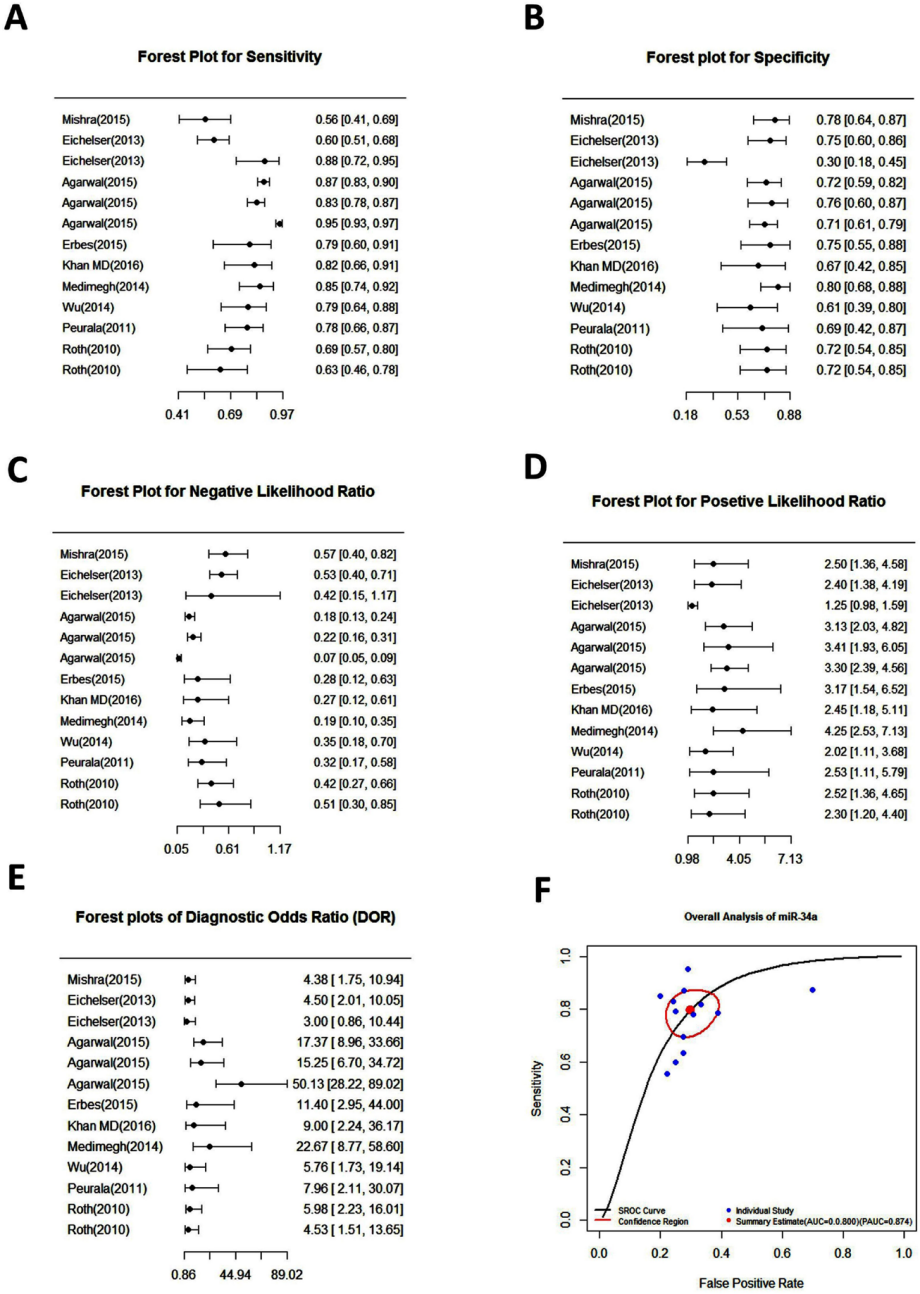
Forest plots of pooled sensitivity (**A**), specificity (**B**), negative likelihood ratio (**C**), positive likelihood ratio (**D**), diagnostic odds ratio (**E**) and summary receiver operating characteristic (SROC) curve (**F**) for miR-34a in the diagnosis of breast cancer.

To assess the heterogeneity from threshold effect, we analyzed the diagnostic threshold with the spearman correlation coefficient. The forest plots of pooled sensitivity, specificity, and diagnostic odds ratio (DOR) with their 95% CIs for individual studies are shown in the Figure 3. The overall pooled results for sensitivity, specificity, negative likelihood ratio (NLR), positive likelihood ratio (PLR), and DOR with their 95% CIs were 85.50% (95%CI: 83.80–87.00%, Figure 3A), 70.00% (95% CI: 65.80-74.10%, Figure 3B), 0.29 (95% CI: 0.19–0.43, Figure 3C), 2.58 (95% CI: 1.94–3.44, Figure 3C), and 9.39 (95% CI: 5.47–16.12, Figure 3E) respectively, which showed that there is no heterogeneity from the threshold effect of sensitivity and specificity (*P* = 0.158). The summary receiver operating characteristic (SROC) curve for the included studies was indicated in Figure 3F with an overall area under the curve (AUC) of 0.8 and a partial AUC of 0.87.

### Subgroup analyses

**Figure 4:**
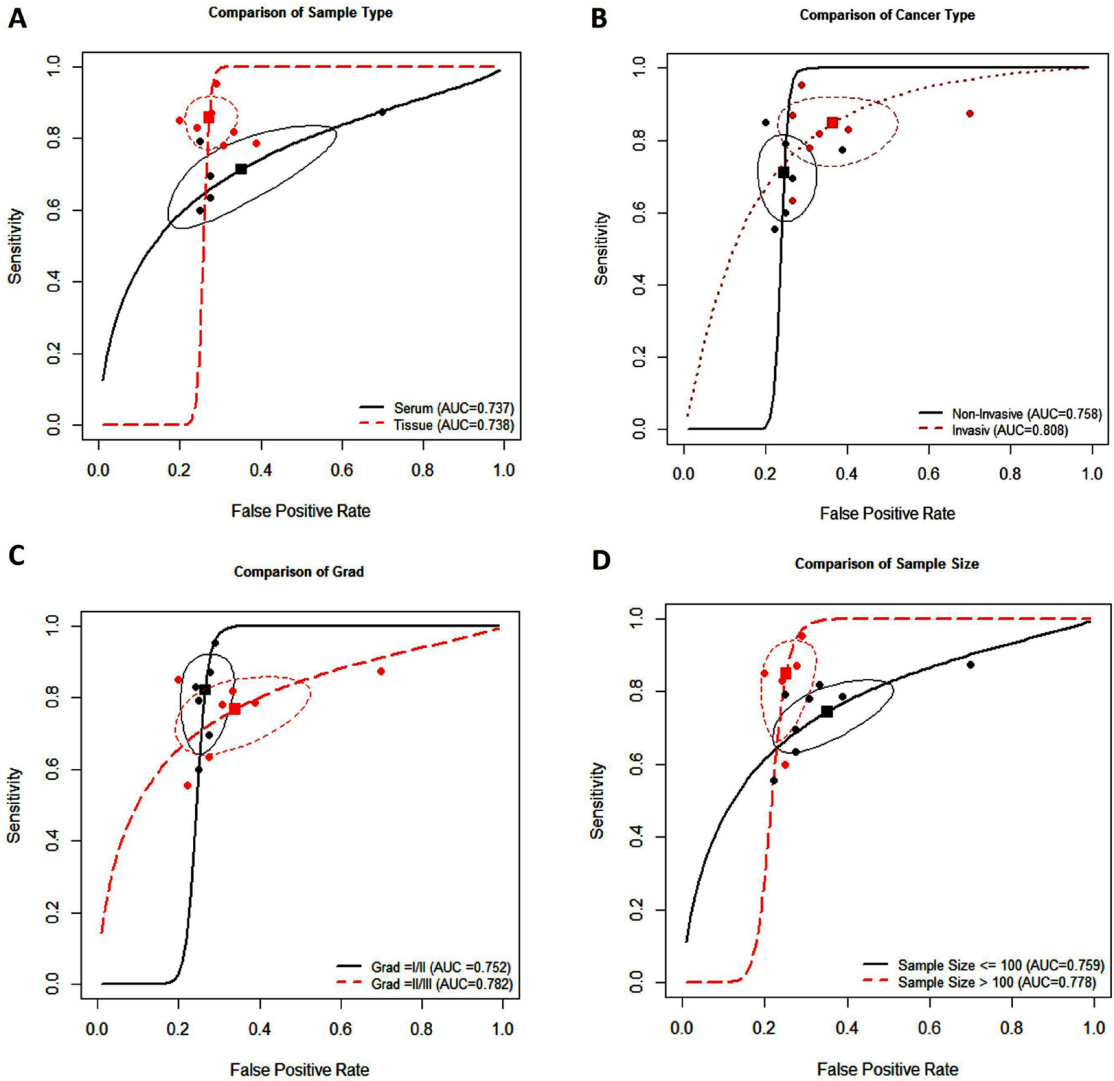
Summary receiver operating characteristic curve for miR-34a and subgroup analysis based on specimen types (**A**), breast cancer type (**B**), breast cancer grade (**C**), and sample size (**D**).

Subgroup analysis was conducted based on ethnicity, cancer type, sample type, sample size (≤ 100 and > 100), RNA measurement method, measurements type, reference control, and cancer grade. Table 3 reveals none of the above covariates contributed to the heterogeneity (all *P* > 0.05). Therefore, on the basis of those covariates, the pooled sensitivity, specificity, AUC, and partial AUC for important sub-analysis parameters were measured. The subgroup analysis based on specimen types indicated that tissue has a relatively accurate diagnostic value in comparison to the serum, with a sensitivity of 0.897 versus 0.675, specificity of 0.728 versus 0.630, and AUC of 0.738 versus 0.737 (Figure 4A).

**Table 3:** Subgroup analyses of the included studies

Subgroup analyses		No.	Sensitivity (95% CI)	Specificity (95% CI)	PLR (95% CI)	NLR (95% CI)	DOR (95% CI)	AUC	PAUC
Ethnicity	Asian	2 (75)	0.800 (0.692–0.884)	0.636 (0.451–0.796)	2.184 (1.373–3.476)	0.316 (0.187–0.532)	6.970 (2.809–17.296)	-	-
	Caucasian	11 (1783)	0.857 (0.840–0.873)	0.705 (0.661–0.746)	2.655 (1.912–3.686)	0.286 (0.183–0.448)	9.754 (5.335–17.833)	0.804	0.879
Cancer type	Non-IBC	6 (350)	0.689 (0.637–0.737)	0.755 (0.692–0.810)	2.768 (2.172–3.325)	0.394 (0.282–0.553)	7.227 (4.194–12.455)	0.758	0.784
	IBC	7 (1508)	0.893 (0.877–0.908)	0.658 (0.599–0.714)	2.486 (1.551–3.985)	0.230 (0.135–0.393)	11.421 (5.250–24.847)	0.808	0.886
Sample type	Tissue	7 (1548)	0.894 (0.878–0.909)	0.728 (0.673–0.779)	3.123 (2.583–3.776)	0.197 (0.125–0.310)	16.304 (8.988–29.572)	0.738	0.678
	Serum	5 (265)	0.675 (0.615–0.731)	0.630 (0.550–0.704)	2.135 (1.304–3.396)	0.478 (0.389–0.587)	5.070 (3.180–8.084)	0.737	0.766
	Plasma	1 (45)	-	-	-	-	-	-	-
Sample size	< = 100	9 (384)	0.735 (0.683–0.782)	0.643 (0.575–0.707)	2.168 (1.536–3.061)	0.430 (0.353–0.525)	5.649 (3.757–8.493)	0.759	0.787
	> 100	4 (1474)	0.880 (0.863–0.896)	0.744 (0.689–0.794)	3.266 (2.672–3.990)	0.194 (0.094–0.398)	17.149 (7.536–39.024)	0.778	0.720
Genotyping method	In situ hyb.	4 (1413)	0.901 (0.884–0.916)	0.722 (0.653–0.783)	3.205 (2.554–4.022)	0.167 (0.089–0.314)	20.141 (9.407–43.125)	0.750	0.816
	qRT-PCR	9 (445)	0.708 (0.663–0.750)	0.687 (0.631–0.739)	2.370 (1.649–3.407)	0.404 (0.313–0.522)	6.429 (4.234–9.763)	0.771	0.798
Ref. control	miR-16	3 (176)	0.676 (0.602–0.745)	0.577 (0.476–0.673)	2.025 (0.965–4.250)	0.455 (0.305–0.678)	4.953 (2.630 -9.326)	0.738	0.772
	U6	4 (180)	0.756 (0.686–0.819)	0.754 (0.673–0.823)	2.789 (1.973–3.944)	0.330 (0.183–0.595)	8.555 (3.823–19.141)	0.782	0.820
	other	6 (1502)	0.887 (0.870–0.903)	0.722 (0.663–0.777)	3.023 (2.469–3.701)	0.233 (0.130–0.418)	12.922 (5.920–28.209)	0.733	0.656
Meas. type	Taqman	8 (385)	0.686 (0.637–0.732)	0.658 (0.595–0.718)	2.162 (1.550–3.015)	0.476 (0.402–0.564)	5.224 (3.580–7.625)	0.744	0.774
	SYBR	1 (60)	-	-	-	-	-	-	-
	NR	4 (1413)	-	-	-	-	-	-	-
Grade	I/II	6 (1557)	0.873 (0.855–0.889)	0.730 (0.673–0.782)	3.053 (2.506–3.718)	0.233 (0.122–0.445)	13.136 (5.866–29.419)	0.752	0.778
	II/III	7 (301)	0.761 (0.709–0.808)	0.664 (0.597–0.726)	2.292 (1.450–3.623)	0.368 (0.263–0.515)	6.763 (3.932–11.632)	0.782	0.804

Abbreviations: IBC, invasive breast cancer; Non-IBC, non- invasive breast cancer; In situ hyb., in situ hybridization; qRT-PCR, quantitative real-time reverse transcription PCR; Meas., measurement; Ref., reference; U6, human U6 snRNA housekeeping small RNA controls; NR, not reported; AUC, area under the curve; PAUC, partial AUC; All study number reported as the number (case number); “NR” parameters for the measurements type and reference control were considered as other categories. The fold changes in miR-34a expression were calculated using the 2^−ΔΔCt^ method. Malignant tumors classified according the tumor-node-metastasis (TNM) stage.

Furthermore, subgroup analysis of different BC types showed highter accuracy of miR-34a in detecting IBC samples (Figure 4B). As shown in Table 3 and Figure 4C, the pooled sensitivity and specificity were higher in grade I/II of BC compared to grade II/III (0.873 versus 0.761, 0.730 versus 0.664, respectively). Meanwhile, highest sensitivity, specificity, AUC, and partial AUC were shown in sample size more than 100, suggesting that miR-34a is more accurate in high sample size diagnosis (Figure 4D).

### Publication bias

**Figure 5:**
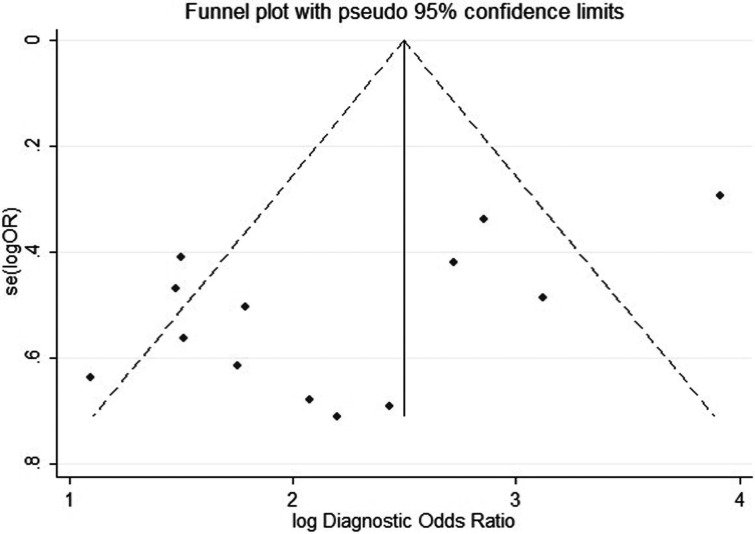
Publication bias by Funnel plot analysis Each point represents a separate study for the indicated association, the vertical axes represent standard error of logarithmic hazard ratio (HR) and horizontal axes represent the HRs limits.

Funnel plots and Begg's test were used to estimate the publication bias, which was carried out repeatedly by precluding a single study at a time (Figure 5) [27]. The resulting shape of the funnel plot and Egger's test provided no statistical evidence for publication bias (*t* = −2.90, *P* = 0.148). Hence, there is no noticeable evidence for significant publication bias in our meta-analysis, which signifies our meta-analysis results were stable and credible.

## DISCUSSION

It is well established that miRNAs have been considered as potential biomarkers in important carcinogenesis processes related to angiogenesis, proliferation, differentiation, invasion, apoptosis, and metastasis. MiRNA's unique expression in cancerous tissue or serum, namely their stable up-regulation of oncogenic miRNAs or down-regulation of tumor suppressive miRNAs has deemed as potential biomarkers for diagnosis of various cancers. MiR-34a, as a potential tumor suppressive miRNA, was significantly down-regulated in variety of solid tumors. MiR-34a is involved in the invasion and migration process of BC by transcriptional regulating p53 network, indicating its potential role as a target for BC detection and therapy [15]. Significantly reduced circulating miR-34a levels in BC patients highlight the potential role of miR-34a as a novel non-invasive biomarker in BC [28]. Recently, Nugent *et al*. reported that miR-34a might be a potential biomarker for BC diagnosis because BC patients have higher serum miR-34a expression than healthy women, making this molecule a promising candidate as a biomarker to reflect various physiological and pathological states of BC [25, 26]. These results suggest that biomarker can serve more use in the clinical settings if it is truly specific to a particular cancer type, which was partially demonstrated in the case of miR-34a.

With these assumptions and foregrounds, we collected all available studies and performed a meta-analysis to confirm the diagnostic value of miR-34a in the detection of BC. We planned to understand the relationship of miR-34a as a diagnostic marker to predict other clinic-pathological features and outcomes of BC, like cancer type, specimen type, cancer grade and sample size. To our best knowledge, no meta-analysis has investigated the association between BC and miR-34a expression by displaying consistent, statistically significant frequency in its expression level.

Conventional histological biomarkers for BC diagnosis, such as estrogen and progesterone receptors, and human epidermal growth factor receptor-2 performances, fall short from perfect diagnostic methods, mainly due to their low diagnostic sensitivity and specificity, high cost and severe invasion [29, 30]. In addition, most computer-based diagnostic methods are rife complexity and instability. Meanwhile, the emergence of new molecular biomarkers, such as neuron-specific enolase [31], carcinoembryonic antigen [32], and cytokeratin-19 fragments [33], cannot be used in clinic settings due to low sensitivity and specificity [34]. Peurala *et al*. showed that low expression of miR-34a was found in about 32% of human breast carcinomas while high expression was in about 25%, with the remaining tumors showing intermediate expression levels [24]. Our meta-analysis showed that vestibular schwannomas are most highly ranked among consistently reported cancer types with down-regulated miR-34a (average FC: 1.43). It is unquestionable that down-regulation of miR-34a was significantly correlated with metastasis and an aggressive phenotype of BC [15, 35]. Our findings also underlined an important association between miR-34a and BC risk (OR = 3.12, 95% CI: 1.83–4.39, *P* < 0.001). Subgroup analysis of cancer types showed significant association between the expression of miR-34a and increased relative risk of IBC (OR = 0.90, 95% CI: 0.877–0.908, *P* = 0.02), well as tissue-based samples (OR = 0.894, 95% CI: 0.878–0.909, *P* = 0.001). Also, a significant diagnostic role of miR-34a was found in large sample sizes than size of less than 100 cases. Our results also suggested miR-34a had more promising accuracy for BC diagnosis in Caucasians than that in Asian patients. It is already well established that miRNA expression profiling might be more precise in the Caucasian population than the Asian population [36]. Considering the limitation of small sample size in the Asian group, further large-size studies among Asian BC population should be designed to provide a comprehensive outcome. Our systematic search clearly indicated that African-American populations, as well as Hispanics, were associated with a risk factor for developing particular forms of BC. ER^+^ BC tends to be associated with Caucasian women, and triple-negative breast cancer tends to be associated with ethnicity/race [37, 38]. MiR-34a is more accurate in a large cohort study of tissue-based IBC. Due to the relatively low overall accuracy based on pooled sensitivity and specificity, the diagnostic accuracy may not be as high as expected. Furthermore, we combined the pooled DOR and SPE data with sensitivity to assay the test accuracy. However, the higher value of DOR represents better test discernment [36].

The assessment of diagnostic accuracy of miR-34a in clinical level was verified by PLR and NLR likelihood ratios test. NLR value of 0.289 (95% CI: 0.19–0.43) indicated that the possibility of the person having BC is around 3% if miR-34a evaluations were negative, which is low enough to rule out cancer. Pooled PLR of 2.58 (95% CI: 1.94–3.43) suggested that BC patients could have about 2.58-fold higher chance of being miR-34a positive compared to healthy control. Specifically, the upper-left corner SROC curve is the perfect test to evaluate diagnostic value [39]. Our overall AUC of SROC is 0.84, indicating miR-34a is highly accurate as a biomarker for BC. Statistically 0.80 AUC and 0.874 partial AUC are considered in good range of SROC (the good range of AUC: 0.75–0.92 [40]).

Sources of interpatient heterogeneity had a critical role in affecting the robustness of meta-analysis results, thus important for calculating the accuracy of miR-34a from randomized samples source. The threshold effect of spearman correlation coefficient is the main reason for heterogeneity in tested accuracy studies [26]. In this study, there was no heterogeneity from the threshold effect with the spearman correlation coefficient of sensitivity and 1-specificity of −0.415 (*P* = 0.148). Even so, we performed meta-regression and subgroup analysis to implement other related factors that affecting heterogeneity. For instance, qRT-PCR was extensively used to test miR-34a expression with the human U6 snRNA as control [41, 42]. Subgroup analysis showed that tissue-based miR-34a had higher accuracy for diagnosing BC [41, 42]. Other results of different subgroups were relatively consistent with the main results, which proposed that our findings are reliable.

We should point out that there are some limitations in this investigation. First, we only included the papers in English language, while published papers in other languages were ignored. Fundamentally, the meta-analysis results were based on unadjusted estimates, because some studies did not provide detailed information to calculate the adjusted estimates. Furthermore, many confounding factors were not controlled or reported in biased statistical results. For example, the unadjusted ORs, specific genetic factors (e.g. BRCA1/2 mutations), and many other clinical factors such as age might lead to bias. In addition, very few African populations (60 cases) were involved in our analysis [22], which may cause selection bias from population. Small sample size, quality of the original studies, and poor homogeneous distribution of the population based on subgroup parameters might be other limitations in our study as well. Well-designed studies in large-scale with matched case-controls and functional studies are of great value to warrant these findings.

## MATERIALS AND METHODS

### Search strategy

A comprehensive systematic search from the literatures published in English was carried out by querying the MEDLINE electronic database, including PubMed, ISI Web of Science, Google Scholar, vendor information pages database, and Embase, to identify all the relevant studies. Based on the research question, the following key words or main heading term were used: “microRNA-34a or micro RNA 34a or miRNA-34a or miR-34a”, “breast or mammary”, “cancer or neoplasm or carcinoma”, and “tumor or tumour”, Alternative spelling and synonyms were incorporated using Boolean “OR” and main terms were linked using Boolean “AND”. All literatures assessed the diagnostic value of miR-34a in BC patients are prior to July 12, 2016, no lower date limit was used. References of articles were also checked for any relevant articles.

### Study inclusion/exclusion criteria

Studies were considered eligible if they met the following criteria: (i) BC was confirmed by histopathological examination; (ii) the levels of miR-34a in tissue or plasma or serum were measured; (iii) the association between the expression level of miR-34a and survival outcomes, like sensitivity, specificity, and cut-off values can be found in each study or measured from the provided data. Exclusion criteria in this meta-analysis were as follows: (i) a review, case-control, conference abstract, meeting, comments, letter or experiment on cell line and animal model; (ii) non English articles; (iii) duplicates or continued works of previous publications; (iv) unqualified key data such as ORs with their 95% CIs, inadequate *P*-value, or useful data calculated by Tierney *et al*. [43], Williamson *et al*. [44], and Parmar *et al*. [45]; (v) articles from one author and the studies with repeated samples from the same patients when a study already included.

### Data extraction

The following key components of all qualified studies were recorded independently by two investigators (XZ and SI): first author's name, publication year, country origin, BC type, characteristics of controls and matching criteria, study design, tumor-node-metastasis stage, tumor size, sampling site, ethnicity, genotyping methods, reference control, RNA extraction, measurement methods, total number of cases and controls, cut-off value, *P*-value for Hardy-Weinberg equilibrium (HWE) of controls, and true and false positives and negatives [43]. Any inconsistencies or disagreements in the research process were resolved through debate and consultations. If they could not reach a consensus, a third partner (JF) resolved these disagreements according to the original data. We also e-mailed the corresponding authors of the selected articles to obtain any missing or additional information and copies of the original data required for the meta-analysis.

### Quality assessment

This present study was performed systematically in accordance with the guidelines of the preferred reporting items for PRISMA [46]. Diagnostic accuracy of studies was validated by QUADAS-2 tool in patient selection, index test, reference standard, and flow timing [47]. QUADAS-2 was assessed to determine the quality of all the studies by three authors (XZ, SI and HH) and any disagreements were resolved through a discussion. Each of the assessment was subjected to seven questions with the answered with “yes”, “no”, or “unclear”. The answer of “yes” means that a study's risk bias can be judged as low, while “no” and “unclear” mean that the risk of bias can be referred as high. The quality assessment table for each selected study is sorted in Supplementary Table 1.

### Statistical analysis

Meta-analysis was performed using Manager Software version 5.2 (software update; The Nordic Cochrane Centre, Copenhagen, Denmark). Data was presented as mean ± Std. deviation (SD) or median (range), including a description of qualitative variables such as number and percentage. Pooled sensitivity, pooled specificity, PLR, NLR, DOR, and corresponding 95% CIs were calculated to evaluate the diagnostic value of miR-34a. HWE was checked by χ^2^ test. The heterogeneity of the combined DOR was evaluated with Cochran's *Q* test and the Higgins I-squared statistic from non-threshold effect. To identify cut-off threshold effects, spearman's rank correlation coefficient test was used to determine associations between two sensitivity and specificity [26]. They were considered statistically heterogeneous if they displayed *P* < 0.05 and/or I2 > 50% [48]. Subgroup analysis was conducted to determine the source of existing heterogeneity. The diagnostic threshold effect was analyzed by the Spearman correlation coefficient test. Additionally, we examined the correlation between miR-34a expression and the clinicopathological variables in BC through OR [49]. Forest plot was used to estimate the diagnostic effects of miR-34a expression on BC diagnosis. Publication bias was evaluated by funnel plot and Egger's regression test [50]. The value less than 0.05 for “Pr > |z|” was considered as potential publication bias [48, 50]. All reported *P* values were two-sided and *P* < 0.05 was considered statistically significant. All statistical analyses were carried out using MetaDiSc version 1.4 and R software (version 3.3.1) packages included “mada” (for sensitivity and specificity analysis).

## CONCLUSIONS

Despite some limitations, the data of the present meta-analysis suggests that miR-34a displays excellent characteristics in BC detection as well as exhibits characteristics of a more accurate diagnostic biomarker in tissue samples of IBC patients. Furthermore, our meta-analysis indicates that miR-34a could be a promising and novel non-invasive biomarker in diagnosing BC.

## SUPPLEMENTARY MATERIALS TABLE


