# Supplementary material for: The diagnostic role of microRNA-34a in breast cancer: a systematic review and meta-analysis
Source: Oncotarget. 2017 Feb 20;8(14):23177–87. doi: 10.18632/oncotarget.15520 (PMC5410295; doi:10.18632/oncotarget.15520)
Supplement: Supplementary file 1 [file oncotarget-08-23177-s001.pdf]

The diagnostic role of microRNA-34a in breast cancer: a systematic review and meta-analysis

Supplementary Materials

Supplementary Table 1: Detailed informationof QUADAS-2 assessment

Study	Risk of bias				Applicability concerns		
	Patient Selection	Index Selection	Reference Standard	Flow and Timing	Patient Selection	Index Selection	Reference Standard
Seema Agarwal [1]	HR	?	LR	LR	LR	LR	LR
Hanna Peurala [2]	LR	?	LR	HR	LR	?	LR
Thalia Erbes [3]	HR	HR	LR	LR	LR	LR	LR
Carina Roth [4]	HR	HR	LR	LR	HR	LR	LR
Sanjay Mishra [5]	HR	?	LR	HR	LR	HR	LR
Imen Medimegh [6]	HR	LR	HR	?	LR	LR	?
Corinna Eichelser [7]	HR	HR	LR	LR	LR	LR	HR
Mei Yi Wu [8]	HR	HR	HR	LR	HR	?	LR
Khan M.A. [9]	HR	HR	LR	HR	HR	LR	LR

Key: LR, Low Risk; HR, High Risk; ?, Unclear Risk.

REFERENCES

1. Agarwal S, Hanna J, Sherman M, Figueroa J, Rimm D. Quantitative assessment of miR34a as an independent prognostic marker in breast cancer. Br J cancer. 2015; 112:61–68.
2. Peurala H, Greco D, Heikkinen T, Kaur S, Bartkova J, Jamshidi M, Aittomaki K, Heikkila P, Bartek J, Blomqvist C, Butzow R, Nevanlinna H. MiR-34a expression has an effect for lower risk of metastasis and associates with expression patterns predicting clinical outcome in breast cancer. PLoS One. 2011; 6:e26122.
3. Erbes T, Hirschfeld M, Rücker G, Jaeger M, Boas J, Iborra S, Mayer S, Gitsch G, Stickeler E. Feasibility of urinary microRNA detection in breast cancer patients and its potential as an innovative non-invasive biomarker. BMC cancer. 2015; 15:1.
4. Roth C, Rack B, Müller V, Janni W, Pantel K, Schwarzenbach H. Circulating microRNAs as blood-based markers for patients with primary and metastatic breast cancer. Breast Cancer Res. 2010; 12:R90.
5. Mishra S, Srivastava AK, Suman S, Kumar V, Shukla Y. Circulating miRNAs revealed as surrogate molecular signatures for the early detection of breast cancer. Cancer Lett. 2015; 369:67–75.
6. Medimegh I, Omrane I, Privat M, Uhrhummer N, Ayari H, Belaiba F, Benayed F, Benromdhan K, Mader S, Bignon IJ, Elgaaied AB. MicroRNAs expression in triple negative vs non triple negative breast cancer in Tunisia: interaction with clinical outcome. PLoS One. 2014; 9:e111877.
7. Eichelser C, Flesch-Janys D, Chang-Claude J, Pantel K, Schwarzenbach H. Deregulated serum concentrations of circulating cell-free microRNAs miR-17, miR-34a, miR-155, and miR-373 in human breast cancer development and progression. Clin Chem. 2013; 59:1489–1496.
8. Wu MY, Fu J, Xiao X, Wu J, Wu RC. MiR-34a regulates therapy resistance by targeting HDAC1 and HDAC7 in breast cancer. Cancer Lett. 2014; 354:311–319.
9. Khan MA, Tania M, Wei C, Fu J. Relationship between transcription factor TWIST1 and microRNA34a in metastatic breast cancer cells. American Association for cancer Research (AACR). (Philadelphia, PA, USA: AACR). 2015.
